# Geographic variations and trends in percutaneous intervention for patients with and without acute myocardial infarction: A Japanese nationwide registry study

**DOI:** 10.1371/journal.pone.0335426

**Published:** 2025-10-31

**Authors:** Yuichi Saito, Yuichiro Mori, Kyohei Yamaji, Shun Kohsaka, Hideki Wada, Hideki Ishii, Tetsuya Amano, Yoshio Kobayashi, Ken Kozuma, for the J-PCI Registry Investigators

**Affiliations:** 1 Department of Cardiovascular Medicine, Chiba University Hospital, Chiba, Japan; 2 Department of Human Health Sciences, Graduate School of Medicine, Kyoto University, Kyoto, Japan; 3 Department of Cardiology, Kyoto University, Kyoto, Japan; 4 Department of Cardiology, Keio University School of Medicine, Tokyo, Japan; 5 Department of Health Data Science, Graduate School of Data Science, Yokohama City University, Yokohama, Japan; 6 Department of Cardiovascular Medicine, Juntendo University Shizuoka Hospital, Izunokuni, Japan; 7 Department of Cardiovascular Medicine, Gunma University Graduate School of Medicine, Gunma, Japan; 8 Department of Cardiology, Aichi Medical University, Nagakute, Japan; 9 Department of Cardiology, Teikyo University Hospital, Tokyo, Japan; Osaka University Graduate School of Medicine, JAPAN

## Abstract

**Background:**

A previous study demonstrated that the per capita volume of percutaneous coronary intervention (PCI) for acute myocardial infarction (AMI) was relatively uniform across the 47 prefectures in Japan, while elective PCIs for stable coronary artery disease showed wide regional variation. However, contemporary data remain limited.

**Methods:**

The Japanese PCI (J-PCI) is a nationwide prospective registry covering most of the procedures performed within the country. PCI procedures in 2019 and 2023 were included and divided according to the indications: AMI versus non-AMI. The patterns of PCI procedures performed for AMI and non-AMI across all prefectures in Japan were evaluated. The associations of the non-AMI/AMI ratio with population, area, and the number of PCI-capable centers per prefecture were also assessed.

**Results:**

A total of 494,746 PCI procedures were analyzed. The ratios between the highest and lowest prefectures were 4.0-fold in non-AMI and 1.9-fold in AMI in 2019 and 4.2-fold in non-AMI and 2.0-fold in AMI in 2023. The non-AMI/AMI ratio was positively correlated with the ratios of the number of PCI-capable centers to population and area per prefecture.

**Conclusions:**

Geographic disparity was observed in the relative volume of PCI performed for AMI compared to non-AMI across Japan, potentially reflecting variations in the density of PCI-capable centers relative to the area and population of each prefecture.

## Introduction

Acute myocardial infarction (AMI) is one of the leading causes of global mortality, and stable coronary artery disease (CAD), including angina pectoris, affects more than 100 million people worldwide, with a projected increase in prevalence [1,2]. In contrast to the declining trend in AMI in the United States and European countries [3,4], the incidence rate has been increasing in East Asian regions such as Japan and China [5,6]. Percutaneous coronary intervention (PCI) is a frequently performed procedure for patients with CAD and has been established to improve the prognosis after AMI [7,8], while the role of elective PCI among patients with stable CAD to reduce the risk of AMI and mortality has been a matter of debate [9,10]. Nonetheless, the number of elective PCI procedures remains stable or has been increasing globally in the past decade [11–13].

Identifying geographic and regional variations to fill the gap of inequity is believed to be central to improving outcomes of patients with CAD undergoing coronary revascularization [14–17]. Currently, Japan is one of the leading countries in healthcare equity in access and quality across the world [18], but regional variations in the management of CAD have been identified within the country [19]. A previous nationwide administrative database study demonstrated that the number of primary PCI procedures for AMI per population was equivalent across 47 prefectures in Japan, while that of elective PCI for angina varied widely with a fivefold ratio between the highest and lowest prefectures in 2013–2014 [20]. Thereafter, several randomized trials have demonstrated no obvious survival benefit of PCI in patients with stable CAD [10,21], and in this context, the Japanese Ministry of Health, Labor and Welfare introduced a new policy to promote the appropriate implementation of elective PCI in 2018, which required the proof of myocardial ischemia for healthcare reimbursement [22]. Nonetheless, it is unclear whether the number of primary PCI relative to elective procedures still varies widely across regions. Using a nationwide registry database, this study aimed to evaluate the patterns of PCI for AMI and non-AMI cases and to identify the underlying mechanisms in contemporary clinical practice across Japan.

## Methods

### Data source

The Japanese PCI (J-PCI) registry is an ongoing, prospective, nationwide registry endorsed by the Japanese Association of Cardiovascular Intervention and Therapeutics (CVIT) [23–26]. The J-PCI registry covers more than 90% of PCI procedures performed across Japan, in which clinical variables and outcome data have been recorded [27]. Individual institutions have a data manager responsible for collecting and reporting data into the web-based database system of the J-PCI. Data registration in the J-PCI registry is mandatory for board certification and renewal by the CVIT. The CVIT has been conducting random audits (20 institutions annually) to assess the quality of registered data. Since 2019, the definition of clinical outcomes of the J-PCI has changed. A third-party central ethics committee (Clinical Research Promotion Network, Osaka, Japan) approved the study protocol for the J-PCI registry. The present study adhered to the Declaration of Helsinki, and written informed consent was waived because of the complete data anonymization of this study.

### Study population and geographical data

**Fig 1:**
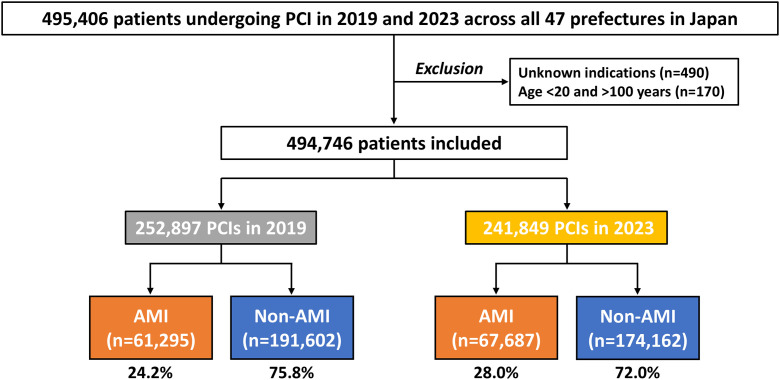
Study flow. AMI, acute myocardial infarction; PCI, percutaneous coronary intervention.

We evaluated the J-PCI data in 2019 and 2023. Patients undergoing PCI procedures across 47 prefectures in Japan were included. Major exclusion criteria included unknown indications for PCI and patients aged <20 or >100 years (Fig 1). PCI procedures were divided into two groups according to the indication of PCI: AMI versus non-AMI, as previously reported (Fig 1) [20]. AMI included both ST-segment elevation and non-ST-segment elevation myocardial infarction [28]. Patient and procedural characteristics, such as age, sex, cardiovascular risk factors, comorbidities, clinical presentations, access site, and coronary stents, were assessed. Geographical data included the area and population in each prefecture. In Japan, a complete census has been conducted every five years to disclose the population status. Geographic population data of the 47 prefectures across Japan were obtained from the census in 2020, provided by the Statistics Bureau, Ministry of Internal Affairs and Communications (data accessed on 22/Apr/2025) [29]. The areas of each prefecture are derived from the Ministry of Land, Infrastructure, Transport and Tourism data in 2024 (data accessed on 22/Apr/2025) [30]. In 2020, Japan was inhabited by approximately 126 million people over a 378,000 km^2^ area across 47 prefectures.

### Outcomes and statistical analysis

The primary interest of this study was to evaluate the patterns of PCI procedures performed for AMI and non-AMI across 47 prefectures in Japan. The relative number of PCI for non-AMI to AMI cases was evaluated with the ratio (i.e., non-AMI/AMI ratio). In addition, potential associations of the non-AMI/AMI ratio with population, area, and the number of PCI-capable centers per prefecture were assessed. PCI-capable centers were defined as institutions where data on PCI procedures were administered in the J-PCI registry in 2019 and 2023. Temporal trends from 2019 to 2023 in the number of PCI procedures for AMI and non-AMI and the non-AMI/AMI ratio were also investigated.

Statistical analyses were performed using R statistical software, version 4.4.3 (R Foundation for Statistical Computing, Vienna, Austria). Data are expressed as the mean ± standard deviation or frequency (percentage). Continuous variables were compared using the Student’s t-test or paired t-test, and categorical data were evaluated using the chi-squared test or Fisher’s exact test. Pearson’s or Spearman’s correlation coefficient was employed to assess associations among continuous variables, based on the normal distribution. The number of PCI procedures for AMI and non-AMI per 100,000 population was adjusted with the distribution of five-year age groups and sex per prefecture using the 2015 Japan Standard Population, following the latest government recommendations [31]. A P value <0.05 was considered statistically significant.

## Results

A total of 494,746 PCI procedures performed in Japan were analyzed in the present study (252,897 in 2019 and 241,849 in 2023) (Fig 1). The proportion of patients with AMI was 24.2% in 2019 and 28.0% in 2023, respectively. Baseline characteristics are listed in Table 1. The mean age and prevalence of cardiovascular risk factors, except for smoking, were increased from 2019 to 2023 in both AMI and non-AMI (Table 1). In-hospital mortality in patients with AMI was 5.5% in 2019 and 6.0% in 2023 (Table 1). Comparable baseline characteristics in 2019 and 2023 are summarized in S1 Table, showing significantly increasing trends in age and the prevalence of comorbidities along with those in the prevalence of AMI and in-hospital mortality during the study periods.

**Table 1:** Baseline characteristics.

Variable	2019		2023	
	AMI	Non-AMI	AMI	Non-AMI
	(n = 61,295)	(n = 191,602)	(n = 67,687)	(n = 174,162)
Age (years)	69.8 ± 12.8	71.4 ± 10.6	70.8 ± 12.7	72.2 ± 10.8
Men	46,583 (76.0%)	146,839 (76.6%)	51537 (76.1%)	134424 (77.2%)
Hypertension	42,148 (68.8%)	147,871 (77.2%)	47,742 (70.5%)	136,883 (78.6%)
Diabetes	22,410 (36.6%)	90,146 (47.0%)	25,987 (38.4%)	84,627 (48.6%)
Dyslipidemia	35,897 (58.6%)	131,275 (68.5%)	40,518 (59.9%)	123,836 (71.1%)
Current smoker	22,438 (36.6%)	53,511 (27.9%)	23,788 (35.1%)	46,299 (26.6%)
Chronic kidney disease	10,943 (17.9%)	42,814 (22.3%)	15,907 (23.5%)	48,543 (27.9%)
Hemodialysis	1,844 (3.0%)	15,591 (8.1%)	2,329 (3.4%)	14,908 (8.6%)
COPD	1,508 (2.5%)	5,140 (2.7%)	2,106 (3.1%)	5,624 (3.2%)
Peripheral artery disease	2,295 (3.7%)	17,376 (9.1%)	2,440 (3.6%)	15,719 (9.0%)
Previous heart failure	4,850 (7.9%)	32,665 (17.0%)	6,490 (9.6%)	35,191 (20.2%)
Previous PCI	9,810 (16.0%)	103,719 (54.1%)	11,347 (16.8%)	91,955 (52.8%)
Previous CABG	871 (1.4%)	7,357 (3.8%)	1,011 (1.5%)	5,997 (3.4%)
PCI access site				
Radial artery	43,295 (70.6%)	142,366 (74.3%)	53,589 (79.2%)	135,742 (77.9%)
Femoral artery	16,131 (26.3%)	37,572 (19.6%)	12,249 (18.1%)	29,108 (16.7%)
Other	1,869 (3.0%)	11,664 (6.1%)	1,849 (2.7%)	9,312 (5.3%)
Drug-eluting stent use	52,405 (85.5%)	157,250 (82.1%)	55,718 (82.3%)	133,748 (76.8%)
In-hospital mortality	3,400 (5.5%)	959 (0.5%)	4,029 (6.0%)	1,031 (0.6%)

AMI, acute myocardial infarction; CABG, coronary artery bypass grafting; COPD, chronic obstructive pulmonary disease; PCI, percutaneous coronary intervention.

**Fig 2:**
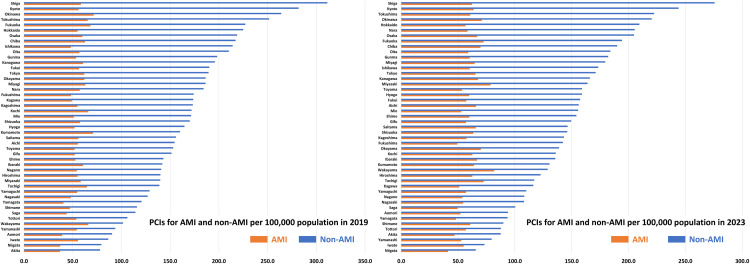
Regional differences in PCI for AMI and non-AMI per population per prefecture in 2019 and 2023. AMI, acute myocardial infarction; PCI, percutaneous coronary intervention.

**Fig 3:**
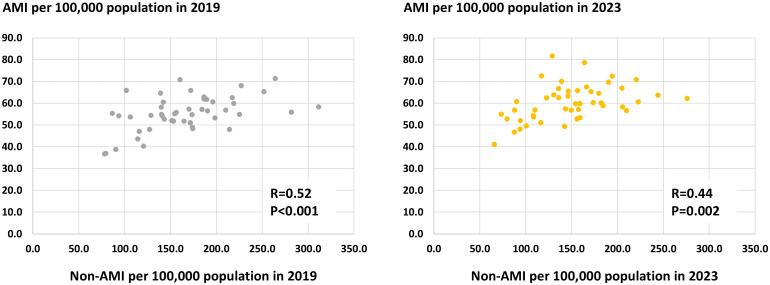
Correlations between the number of percutaneous coronary intervention procedures for AMI and non-AMI. Correlations were assessed using Pearson’s correlation coefficient because of the normal distribution. AMI, acute myocardial infarction.

**Fig 4:**
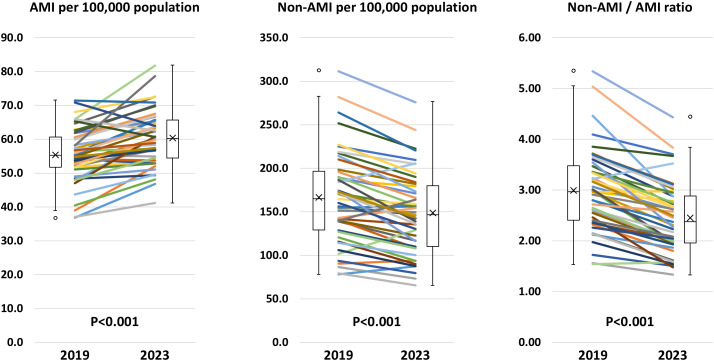
Temporal changes in AMI, non-AMI, and the non-AMI/AMI ratio per prefecture from 2019 to 2023. AMI, acute myocardial infarction.

**Fig 5:**
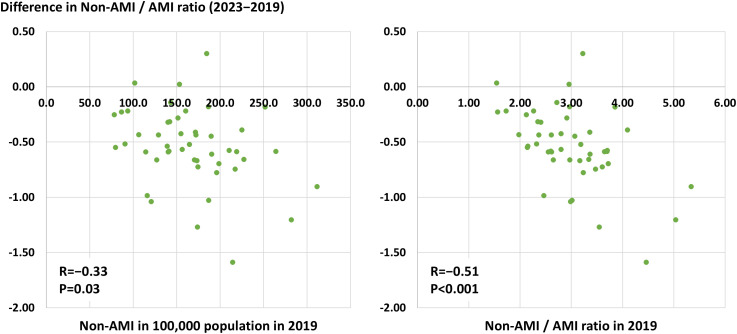
Correlations between the difference in the non-AMI/AMI ratio and the number of non-AMI per population or the non-AMI/AMI ratio in 2019. The differences in the non-AMI/AMI ratio were calculated as the ratio in 2023 minus that in 2019. Correlations were assessed using Pearson’s correlation coefficient because of the normal distribution. AMI, acute myocardial infarction.

**Fig 6:**
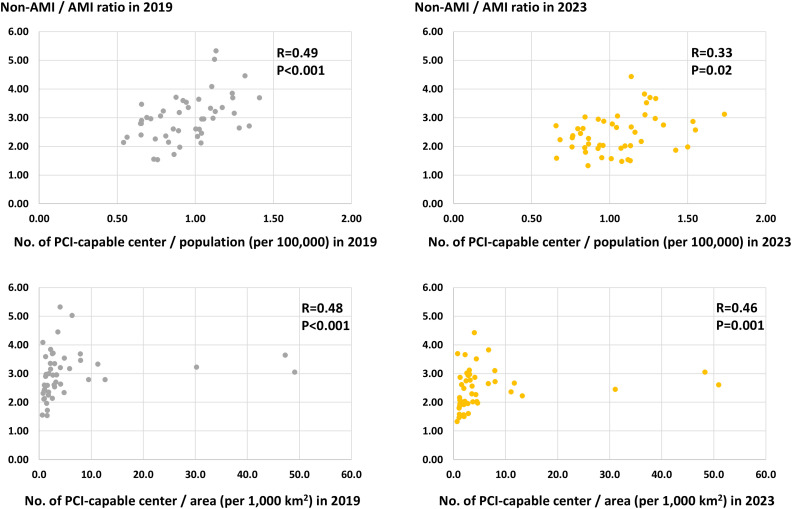
Correlations between the non-AMI/AMI ratio with the ratio of population and area to the number of PCI-capable centers per prefecture. Correlations were assessed using Spearman’s rank correlation. This non-parametric test was chosen because of the outliers (apparent in the lower panels). AMI, acute myocardial infarction; PCI, percutaneous coronary intervention.

Geographic characteristics per prefecture, such as area, population, and the number of PCI-capable centers are shown in S2 Table. The number of PCI procedures for AMI and non-AMI per 100,000 population per prefecture in 2019 and 2023 are illustrated in Fig 2. The crude and adjusted numbers are summarized in S3 and S4 Table. The volume of PCI procedures for non-AMI was greater than that for AMI in all 47 prefectures in 2019 and 2023. The ratios between the highest and lowest prefectures were 4.0-fold in non-AMI (311.3 cases in Shiga prefecture and 78.0 cases in Akita prefecture per 100,000 population) and 1.9-fold in AMI (71.4 cases in Okinawa prefecture and 36.8 cases in Akita prefecture per 100,000 population) in 2019 and 4.2-fold in non-AMI (275.8 cases in Shiga prefecture and 65.6 cases in Niigata prefecture per 100,000 population) and 2.0-fold in AMI (81.8 cases in Wakayama prefecture and 41.2 cases in Niigata prefecture per 100,000 population) in 2023. Significantly positive correlations between the number of PCI procedures for non-AMI and AMI per population per prefecture were observed in 2019 and 2023 (Fig 3). From 2019 to 2023, the number of AMI per 100,000 population per prefecture increased (55.3 ± 8.1 vs. 60.4 ± 8.2, P < 0.001), while that of non-AMI decreased (166.2 ± 82.7 vs. 148.5 ± 46.7, P < 0.001) (Fig 4). Hence, the non-AMI/AMI ratio per prefecture significantly decreased from 2.99 ± 0.80 to 2.45 ± 0.69 during the study period (Fig 4). The difference in the non-AMI/AMI ratio between 2019 and 2023 was negatively correlated with the number of PCI procedures for non-AMI and the ratio in 2019, indicating that the non-AMI/AMI ratio was more likely to decrease in prefectures with a higher elective PCI volume at baseline (in 2019) (Fig 5). We found significantly positive correlations of the non-AMI/AMI ratio with the ratio of the number of PCI-capable centers to population and area per prefecture in both 2019 and 2023 (Fig 6).

## Discussion

The present study from nationwide registry data demonstrated that PCI procedures for non-AMI cases are consistently more frequently performed than those for AMI in Japan, although the relative number of PCI procedures for non-AMI to AMI decreased from 2019 to 2023. The number of PCI procedures for AMI per population was relatively equivalent across 47 prefectures in Japan, whereas that of elective PCI for non-AMI varied widely, with an approximately fourfold ratio between the highest and lowest prefectures. The higher non-AMI/AMI ratio was associated with the greater number of PCI-capable centers per population and area, suggesting that an excess in supply of PCI capability may result in an increased elective PCI volume.

### PCIs for AMI and non-AMI

Primary PCI is an established procedure in patients with AMI, particularly in those with ST-segment elevation myocardial infarction, to improve survival [7,8]. To date, on the other hand, no pivotal randomized trials have shown the survival benefit of PCI over optimal medical therapy in patients with stable CAD, although the risk of spontaneous AMI is potentially reduced by the upfront invasive procedures [10]. Thus, from a public health perspective, it is conceivable that PCI procedures for AMI are more valuable than those for non-AMI [8,32]. In Japan, however, elective PCI procedures for non-AMI have been frequently performed as compared to other countries. In the United States, approximately two-thirds of PCI procedures were performed in a non-elective setting, while less than 30% were non-elective procedures in Japan from 2013 to 2017 [11]. Thus, elective PCI procedures are more than twice as common in Japan as in the United States in contemporary practice, while several randomized trials published over the past decade have questioned the clinical relevance of elective intervention for stable CAD [9,10,21]. In this context, the Japanese Ministry of Health, Labor and Welfare introduced a new policy in 2018 to promote the appropriate implementation of elective PCI, which required proof of myocardial ischemia for healthcare reimbursement [22]. Subsequently, the Japanese professional society, CVIT, proposed the institutional quality indicators in 2019, in which the proportion of PCI procedures for acute coronary syndrome (ACS) was included [33]. Over the dynamic changes in the environment surrounding elective PCI procedures for non-AMI, contemporary data on the pattern of PCI procedures for AMI and non-AMI, with geographical disparities, are warranted.

### Geographical variations in PCI

Using a large-scale administrative database from 2013 to 2014, Inoue et al. reported that the number of PCI procedures for angina and AMI per 100,000 people was 189 and 67 in Japan [20]. In their study, the ratios between the highest and lowest prefectures were 4.9-fold in angina and 1.8-fold in AMI [20], which is in line with the present study findings. Although a geographic variation in AMI, represented by the ratios between the highest and lowest prefectures of approximately twofold, may also be clinically relevant, the ratio was higher in non-AMI. The greater disparity in non-AMI rather than in AMI was also found in the implementation pattern in coronary catheterization in Germany [34]. While the number of PCI procedures for AMI has increased, probably because of the aging society, widespread use of high-sensitivity troponin, and upcoding [35,36], and that for non-AMI has decreased from 2019 to 2023, the ratios evaluating the geographical variation in primary and elective PCI procedures in the report by Inoue et al. closely fit our results [20], suggesting that the practice patterns within Japan remain overall unchanged even after the dynamic change during the past decade. Potential mechanisms of the high elective PCI volume in some prefectures include the relatively greater number of PCI-capable centers per population and area. When the number of PCI-capable centers is higher per population in a prefecture, the non-AMI/AMI ratio was likely to increase. Similarly, when an institution covered a relatively smaller area in a prefecture, the non-AMI/AMI ratio became higher. The excess supply appears to lead to the greater use of PCI procedures for more discretionary indications, such as in patients with stable CAD who may not need or benefit from the elective procedures [37]. Thus, indications and standards for PCI procedures for non-AMI might differ according to density of PCI-capable centers. Although the optimal cut-off value of the non-AMI/AMI ratio is unestablished and the ratio decreased from 2019 to 2023, the fact that the non-AMI/AMI ratio was beyond one in all 47 prefectures in both years reinforces further considerations for elective PCI use. The primary-to-elective PCI ratio at the hospital level has been established as a quality indicator of mortality after AMI [38,39]. Notably, however, greater access to PCI procedures should be beneficial in a setting of AMI [37]. Our results highlight the challenges to balance and optimize the distribution of PCI-capable centers. We believe that the present study findings can provide insights into future medical policies to develop sustainable and effective healthcare systems in CAD and PCI in Japan and other regions.

### Study limitations

This study had some limitations. In the present study, the PCI indications were divided into AMI versus non-AMI rather than ACS versus non-ACS. Thus, a direct comparison to previous studies in this context may be challenging, in which unstable angina was included in the ACS group. The institutional location where PCI procedures were performed did not necessarily correspond to the residence of patients in the same prefecture. In addition, staged PCI procedures following AMI, which reportedly contribute to better outcomes as compared with those for stable CAD, were included in the non-AMI group in this study [8]. Although the greater number of PCI-capable centers relative to the population and area per prefecture suggested a higher non-AMI/AMI ratio, the direct cause-and-effect relationship remains to be established because of the observational study nature. For instance, it is possible that in prefectures with the greater volume of PCI-capable centers, people are likely to be health-conscious and have easier access to medical facilities, potentially leading to early diagnosis of stable CAD and elective PCI procedures. In this study in Japan, where a PCI-to-coronary artery bypass grafting ratio is high [40], the impact of surgical revascularization on the pattern of PCI procedures was not evaluated. Additionally, the present study evaluated PCI volumes in 2019 and 2023, during which the pandemic of coronavirus disease 2019 developed. The first patient with coronavirus disease 2019 was reported in January 2020 in Japan [27], and it is conceivable that the practice pattern was almost normalized in 2023. However, the pandemic may have impacted the results of this study.

## Conclusions

In Japan, where the PCI volume for non-AMI cases is greater than that for AMI, the number of PCI procedures for AMI per population was relatively equivalent across 47 prefectures, while the elective PCI volume varied widely, with a fourfold ratio between the highest and lowest prefectures. Despite the decreasing trend, the non-AMI/AMI ratio remains high. The higher density of PCI-capable centers relative to population and area may be attributable to the excess in PCI procedures for non-AMI. Our results can be a benchmark for healthcare professionals, policymakers, and the public when discussing appropriate medical schemes and the geographical distribution of institutions for CAD and PCI.

## Supporting information

S1 Table Baseline characteristics in 2019 and 2023. (DOCX)

S2 Table Geographic characteristics per prefecture across Japan. (DOCX)

S3 Table The number of PCI procedures per prefecture. (DOCX)

S4 Table Age- and sex-adjusted number of PCIs per prefecture per 100,000 population. (DOCX)
