# Supplementary material for: Geographic variations and trends in percutaneous intervention for patients with and without acute myocardial infarction: A Japanese nationwide registry study
Source: PLoS One. 2025 Oct 31;20(10):e0335426. doi: 10.1371/journal.pone.0335426 (PMC12578162; doi:10.1371/journal.pone.0335426)
Supplement: S1 Table — (DOCX) [file pone.0335426.s001.docx]

**Table S1. Baseline characteristics in 2019 and 2023**

| Variable | 2019  (n=252,897) | 2023  (n=241,849) | P value |
| --- | --- | --- | --- |
| Age (years) | 71.0±11.2 | 71.8±11.4 | <0.001 |
| Men | 193,422 (76.5%) | 185,961 (76.9%) | 0.001 |
| Hypertension | 190,019 (75.1%) | 184,625 (76.3%) | <0.001 |
| Diabetes | 112,556 (44.5%) | 110,614 (45.7%) | <0.001 |
| Dyslipidemia | 167,172 (66.1%) | 164,354 (68.0%) | <0.001 |
| Current smoker | 75,949 (30.0%) | 70,087 (29.0%) | <0.001 |
| Chronic kidney disease | 53,757 (21.3%) | 64,450 (26.6%) | <0.001 |
| Hemodialysis | 17,435 (6.9%) | 17,237 (7.1%) | <0.001 |
| COPD | 6,648 (2.6%) | 7,730 (3.2%) | <0.001 |
| Peripheral artery disease | 19,671 (7.8%) | 18,159 (7.5%) | <0.001 |
| Previous heart failure | 37,515 (14.8%) | 41,681 (17.2%) | <0.001 |
| Previous PCI | 113,529 (44.9%) | 103,302 (42.7%) | <0.001 |
| Previous CABG | 8,228 (3.3%) | 7,008 (2.9%) | <0.001 |
| Indication of PCI |  |  | <0.001 |
| AMI | 61,295 (24.2%) | 67,687 (28.0%) |  |
| Non-AMI | 191,602 (75.8%) | 174,162 (72.0%) |  |
| PCI access site |  |  | <0.001 |
| Radial artery | 53,703 (21.2%) | 41,357 (17.1%) |  |
| Femoral artery | 185,661 (73.4%) | 189,331 (78.3%) |  |
| Other | 13,533 (5.4%) | 11,161 (4.6%) |  |
| Drug-eluting stent use | 209,655 (82.9%) | 189,466 (78.3%) | <0.001 |
| In-hospital mortality | 4,359 (1.7%) | 5,060 (2.1%) | <0.001 |

AMI, acute myocardial infarction; CABG, coronary artery bypass grafting; COPD, chronic obstructive pulmonary disease; PCI, percutaneous coronary intervention.
