# Supplementary material for: Geographic variations and trends in percutaneous intervention for patients with and without acute myocardial infarction: A Japanese nationwide registry study
Source: PLoS One. 2025 Oct 31;20(10):e0335426. doi: 10.1371/journal.pone.0335426 (PMC12578162; doi:10.1371/journal.pone.0335426)
Supplement: S2 Table — (DOCX) [file pone.0335426.s002.docx]

**Table S2. Geographic characteristics per prefecture across Japan**

| Variable | Area  (km^2^) |  | Population (thousand) | |  | PCI-capable center | |
| --- | --- | --- | --- | --- | --- | --- | --- |
|  |  |  | 2019 | 2023 |  | 2019 | 2023 |
| Japan | 377,976 |  | 126,167 | 124,352 |  | 1,113 | 1,181 |
| Hokkaido | 83,422 |  | 5,250 | 5,092 |  | 58 | 64 |
| Aomori | 9,645 |  | 1,246 | 1,184 |  | 7 | 10 |
| Iwate | 15,275 |  | 1,227 | 1,163 |  | 9 | 10 |
| Miyagi | 7,282 |  | 2,306 | 2,264 |  | 24 | 23 |
| Akita | 1,1638 |  | 966 | 914 |  | 10 | 13 |
| Yamagata | 9,323 |  | 1,078 | 1,026 |  | 12 | 11 |
| Fukushima | 13,784 |  | 1,846 | 1,767 |  | 17 | 17 |
| Ibaraki | 6,098 |  | 2,860 | 2,825 |  | 29 | 27 |
| Tochigi | 6,408 |  | 1,934 | 1,897 |  | 16 | 18 |
| Gunma | 6,362 |  | 1,942 | 1,902 |  | 17 | 16 |
| Saitama | 3,798 |  | 7,350 | 7,331 |  | 48 | 50 |
| Chiba | 5,156 |  | 6,259 | 6,257 |  | 41 | 41 |
| Tokyo | 2,200 |  | 13,921 | 14,086 |  | 108 | 112 |
| Kanagawa | 2,417 |  | 9,198 | 9,229 |  | 73 | 75 |
| Niigata | 12,584 |  | 2,223 | 2,126 |  | 12 | 14 |
| Toyama | 4,248 |  | 1,044 | 1,007 |  | 11 | 13 |
| Ishikawa | 4,186 |  | 1,138 | 1,109 |  | 15 | 17 |
| Fukui | 4,191 |  | 768 | 744 |  | 9 | 10 |
| Yamanashi | 4,465 |  | 811 | 796 |  | 7 | 9 |
| Nagano | 13,562 |  | 2,049 | 2,004 |  | 21 | 22 |
| Gifu | 10,621 |  | 1,987 | 1,931 |  | 13 | 16 |
| Shizuoka | 7,777 |  | 3,644 | 3,555 |  | 26 | 27 |
| Aichi | 5,173 |  | 7,552 | 7,477 |  | 49 | 57 |
| Mie | 5,774 |  | 1,781 | 1,727 |  | 17 | 16 |
| Shiga | 4,017 |  | 1,414 | 1,407 |  | 16 | 16 |
| Kyoto | 4,612 |  | 2,583 | 2,535 |  | 29 | 31 |
| Osaka | 1,905 |  | 8,809 | 8,763 |  | 90 | 92 |
| Hyogo | 8,401 |  | 5,466 | 5,370 |  | 49 | 56 |
| Nara | 3,691 |  | 1,330 | 1,296 |  | 15 | 16 |
| Wakayama | 4,725 |  | 925 | 892 |  | 7 | 9 |
| Tottori | 3,507 |  | 556 | 537 |  | 5 | 6 |
| Shimane | 6,708 |  | 674 | 650 |  | 7 | 7 |
| Okayama | 7,114 |  | 1,890 | 1,847 |  | 13 | 14 |
| Hiroshima | 8,478 |  | 2,804 | 2,738 |  | 25 | 23 |
| Yamaguchi | 6,113 |  | 1,358 | 1,298 |  | 11 | 12 |
| Tokushima | 4,147 |  | 728 | 695 |  | 9 | 9 |
| Kagawa | 1,877 |  | 956 | 926 |  | 9 | 8 |
| Ehime | 5,676 |  | 1,339 | 1,291 |  | 18 | 20 |
| Kochi | 7,102 |  | 698 | 666 |  | 7 | 8 |
| Fukuoka | 4,988 |  | 5,104 | 5,103 |  | 56 | 58 |
| Saga | 2,441 |  | 815 | 795 |  | 7 | 9 |
| Nagasaki | 4,131 |  | 1,327 | 1,267 |  | 17 | 19 |
| Kumamoto | 7,409 |  | 1,748 | 1,709 |  | 13 | 16 |
| Oita | 6,341 |  | 1,135 | 1,096 |  | 16 | 19 |
| Miyazaki | 7,734 |  | 1,073 | 1,042 |  | 7 | 9 |
| Kagoshima | 9,186 |  | 1,602 | 1,549 |  | 20 | 18 |
| Okinawa | 2,282 |  | 1,453 | 1,468 |  | 18 | 18 |

Areas of each prefecture are derived from the Ministry of Land, Infrastructure, Transport and Tourism data in 2024.^30^ Populations of each prefecture are derived from the Ministry of Internal Affairs and Communications data.^29^
