# Supplementary material for: Geographic variations and trends in percutaneous intervention for patients with and without acute myocardial infarction: A Japanese nationwide registry study
Source: PLoS One. 2025 Oct 31;20(10):e0335426. doi: 10.1371/journal.pone.0335426 (PMC12578162; doi:10.1371/journal.pone.0335426)
Supplement: S3 Table — (DOCX) [file pone.0335426.s003.docx]

**Table S3. The number of PCI procedures per prefecture**

| Variable | 2019 | | |  | 2023 | | |
| --- | --- | --- | --- | --- | --- | --- | --- |
|  | AMI  (n=61,295) | Non-AMI  (n=191,602) | Non-AMI/AMI ratio |  | AMI  (n=67,687) | Non-AMI  (n=174,162) | Non-AMI/AMI ratio |
| Hokkaido | 2,645 | 11,064 | 4.18 |  | 2,750 | 10,400 | 3.78 |
| Aomori | 460 | 1,073 | 2.33 |  | 617 | 1,137 | 1.84 |
| Iwate | 637 | 987 | 1.55 |  | 642 | 851 | 1.33 |
| Miyagi | 1,215 | 3,593 | 2.96 |  | 1,255 | 3,485 | 2.78 |
| Akita | 365 | 763 | 2.09 |  | 474 | 888 | 1.87 |
| Yamagata | 411 | 1,238 | 3.01 |  | 490 | 966 | 1.97 |
| Fukushima | 815 | 2,959 | 3.63 |  | 829 | 2,433 | 2.93 |
| Ibaraki | 1,507 | 3,560 | 2.36 |  | 1,692 | 3,454 | 2.04 |
| Tochigi | 1,074 | 2,325 | 2.16 |  | 1,211 | 1,979 | 1.63 |
| Gunma | 916 | 3,434 | 3.75 |  | 1,042 | 3,187 | 3.06 |
| Saitama | 3,359 | 9,437 | 2.81 |  | 3,944 | 8,922 | 2.26 |
| Chiba | 3,283 | 11,402 | 3.47 |  | 3,649 | 10,041 | 2.75 |
| Tokyo | 6,391 | 19,062 | 2.98 |  | 6,763 | 17,328 | 2.56 |
| Kanagawa | 4,485 | 14,328 | 3.19 |  | 5,000 | 12,270 | 2.45 |
| Niigata | 770 | 1,629 | 2.12 |  | 861 | 1,365 | 1.59 |
| Toyama | 502 | 1,499 | 2.99 |  | 512 | 1,583 | 3.09 |
| Ishikawa | 473 | 2,115 | 4.47 |  | 597 | 1,724 | 2.89 |
| Fukui | 393 | 1,300 | 3.31 |  | 396 | 1,092 | 2.76 |
| Yamanashi | 399 | 687 | 1.72 |  | 391 | 594 | 1.52 |
| Nagano | 1,035 | 2,692 | 2.60 |  | 1,026 | 2,085 | 2.03 |
| Gifu | 912 | 2,710 | 2.97 |  | 1,014 | 2,687 | 2.65 |
| Shizuoka | 1,840 | 5,495 | 2.99 |  | 2,053 | 4,763 | 2.32 |
| Aichi | 3,248 | 9,097 | 2.80 |  | 3,896 | 9,266 | 2.38 |
| Mie | 797 | 2,690 | 3.38 |  | 836 | 2,482 | 2.97 |
| Shiga | 655 | 3,507 | 5.35 |  | 705 | 3,138 | 4.45 |
| Kyoto | 1,254 | 6,348 | 5.06 |  | 1,416 | 5,546 | 3.92 |
| Osaka | 4,386 | 16,185 | 3.69 |  | 4,939 | 15,219 | 3.08 |
| Hyogo | 2,466 | 7,934 | 3.22 |  | 2,870 | 7,700 | 2.68 |
| Nara | 701 | 2,279 | 3.25 |  | 714 | 2,567 | 3.60 |
| Wakayama | 587 | 904 | 1.54 |  | 730 | 1,171 | 1.60 |
| Tottori | 280 | 546 | 1.95 |  | 292 | 458 | 1.57 |
| Shimane | 308 | 762 | 2.47 |  | 395 | 581 | 1.47 |
| Okayama | 1,030 | 3,112 | 3.02 |  | 1,178 | 2,341 | 1.99 |
| Hiroshima | 1,331 | 3,405 | 2.56 |  | 1,531 | 2,996 | 1.96 |
| Yamaguchi | 709 | 1,704 | 2.40 |  | 752 | 1,450 | 1.93 |
| Tokushima | 450 | 1,756 | 3.90 |  | 416 | 1,565 | 3.76 |
| Kagawa | 423 | 1,528 | 3.61 |  | 438 | 1,016 | 2.32 |
| Ehime | 671 | 1,827 | 2.72 |  | 758 | 1,965 | 2.59 |
| Kochi | 466 | 1,204 | 2.58 |  | 444 | 973 | 2.19 |
| Fukuoka | 2,900 | 9,637 | 3.32 |  | 3,091 | 8,294 | 2.68 |
| Saga | 309 | 822 | 2.66 |  | 354 | 718 | 2.03 |
| Nagasaki | 588 | 1,575 | 2.68 |  | 683 | 1,358 | 1.99 |
| Kumamoto | 1,118 | 2,519 | 2.25 |  | 1,024 | 2,078 | 2.03 |
| Oita | 600 | 2,228 | 3.71 |  | 629 | 1,990 | 3.16 |
| Miyazaki | 570 | 1,390 | 2.44 |  | 785 | 1,659 | 2.11 |
| Kagoshima | 807 | 2,565 | 3.18 |  | 854 | 2,131 | 2.50 |
| Okinawa | 754 | 2,726 | 3.62 |  | 749 | 2,266 | 3.03 |

AMI, acute myocardial infarction; PCI, percutaneous coronary intervention.
