# Supplementary material for: Geographic variations and trends in percutaneous intervention for patients with and without acute myocardial infarction: A Japanese nationwide registry study
Source: PLoS One. 2025 Oct 31;20(10):e0335426. doi: 10.1371/journal.pone.0335426 (PMC12578162; doi:10.1371/journal.pone.0335426)
Supplement: S4 Table — (DOCX) [file pone.0335426.s004.docx]

**Table S4.** **Age- and sex-adjusted number of PCIs per prefecture per 100,000 population**

| Variable | 2019 | | |  | 2023 | | |
| --- | --- | --- | --- | --- | --- | --- | --- |
|  | AMI | Non-AMI | Non-AMI/AMI ratio |  | AMI | Non-AMI | Non-AMI/AMI ratio |
| Hokkaido | 54.9 | 224.9 | 4.09 |  | 56.6 | 209.5 | 3.70 |
| Aomori | 39.0 | 90.4 | 2.32 |  | 52.1 | 93.9 | 1.80 |
| Iwate | 55.4 | 86.5 | 1.56 |  | 54.9 | 73.3 | 1.33 |
| Miyagi | 62.9 | 186.3 | 2.96 |  | 64.5 | 179.5 | 2.78 |
| Akita | 36.8 | 78.0 | 2.12 |  | 46.8 | 87.5 | 1.87 |
| Yamagata | 40.4 | 120.6 | 2.98 |  | 48.1 | 93.6 | 1.94 |
| Fukushima | 48.3 | 174.1 | 3.60 |  | 49.4 | 142.2 | 2.88 |
| Ibaraki | 60.5 | 141.9 | 2.35 |  | 66.7 | 135.6 | 2.03 |
| Tochigi | 64.6 | 138.9 | 2.15 |  | 72.5 | 117.0 | 1.61 |
| Gunma | 53.3 | 198.3 | 3.72 |  | 60.2 | 182.1 | 3.03 |
| Saitama | 55.8 | 156.1 | 2.80 |  | 65.6 | 146.4 | 2.23 |
| Chiba | 62.6 | 217.2 | 3.47 |  | 69.7 | 190.1 | 2.73 |
| Tokyo | 61.8 | 189.4 | 3.06 |  | 65.3 | 171.1 | 2.62 |
| Kanagawa | 60.6 | 195.9 | 3.23 |  | 67.5 | 166.0 | 2.46 |
| Niigata | 37.1 | 79.4 | 2.14 |  | 41.2 | 65.6 | 1.59 |
| Toyama | 51.8 | 153.1 | 2.96 |  | 53.4 | 159.1 | 2.98 |
| Ishikawa | 48.0 | 214.1 | 4.46 |  | 60.3 | 173.3 | 2.88 |
| Fukui | 56.5 | 190.1 | 3.36 |  | 57.2 | 157.5 | 2.75 |
| Yamanashi | 54.2 | 93.6 | 1.73 |  | 52.8 | 79.6 | 1.51 |
| Nagano | 54.2 | 140.8 | 2.60 |  | 53.7 | 108.3 | 2.02 |
| Gifu | 52.0 | 151.3 | 2.91 |  | 56.9 | 149.5 | 2.63 |
| Shizuoka | 57.3 | 170.2 | 2.97 |  | 63.3 | 146.0 | 2.31 |
| Aichi | 55.3 | 154.6 | 2.80 |  | 65.8 | 156.2 | 2.37 |
| Mie | 51.1 | 171.4 | 3.36 |  | 52.8 | 155.6 | 2.95 |
| Shiga | 58.3 | 311.3 | 5.34 |  | 62.2 | 275.8 | 4.43 |
| Kyoto | 55.9 | 281.8 | 5.04 |  | 63.7 | 244.0 | 3.83 |
| Osaka | 59.9 | 218.7 | 3.65 |  | 66.9 | 204.8 | 3.06 |
| Hyogo | 51.7 | 164.6 | 3.18 |  | 59.8 | 159.1 | 2.66 |
| Nara | 57.2 | 184.3 | 3.22 |  | 58.3 | 205.4 | 3.52 |
| Wakayama | 65.9 | 101.6 | 1.54 |  | 81.8 | 128.8 | 1.58 |
| Tottori | 53.8 | 106.1 | 1.97 |  | 56.9 | 87.7 | 1.54 |
| Shimane | 47.1 | 115.9 | 2.46 |  | 60.8 | 89.9 | 1.48 |
| Okayama | 62.0 | 186.5 | 3.01 |  | 70.0 | 138.9 | 1.98 |
| Hiroshima | 54.9 | 140.0 | 2.55 |  | 62.5 | 122.6 | 1.96 |
| Yamaguchi | 54.5 | 128.8 | 2.36 |  | 57.0 | 110.0 | 1.93 |
| Tokushima | 65.3 | 251.6 | 3.85 |  | 60.7 | 222.5 | 3.67 |
| Kagawa | 49.0 | 173.7 | 3.55 |  | 51.1 | 116.2 | 2.28 |
| Ehime | 52.7 | 143.1 | 2.71 |  | 59.8 | 154.1 | 2.57 |
| Kochi | 65.9 | 171.9 | 2.61 |  | 62.6 | 136.0 | 2.17 |
| Fukuoka | 68.0 | 227.1 | 3.34 |  | 72.4 | 194.1 | 2.68 |
| Saga | 43.7 | 114.3 | 2.61 |  | 49.5 | 100.3 | 2.03 |
| Nagasaki | 48.0 | 126.9 | 2.65 |  | 54.5 | 108.0 | 1.98 |
| Kumamoto | 70.8 | 160.1 | 2.26 |  | 63.9 | 130.5 | 2.04 |
| Oita | 56.8 | 210.2 | 3.70 |  | 58.9 | 184.0 | 3.13 |
| Miyazaki | 58.2 | 139.8 | 2.40 |  | 78.6 | 163.9 | 2.08 |
| Kagoshima | 54.8 | 173.2 | 3.16 |  | 57.4 | 143.2 | 2.50 |
| Okinawa | 71.4 | 264.0 | 3.69 |  | 70.9 | 220.4 | 3.11 |

The number of PCI procedures for AMI and non-AMI per 100,000 population was adjusted with the distribution of age and sex per prefecture.^31^ AMI, acute myocardial infarction; PCI, percutaneous coronary intervention.
